# A Self-Healing Gel Polymer Electrolyte, Based on a Macromolecule Cross-Linked Chitosan for Flexible Supercapacitors

**DOI:** 10.3390/gels9010008

**Published:** 2022-12-23

**Authors:** Xiaoyuan Xue, Long Wan, Wenwen Li, Xueling Tan, Xiaoyu Du, Yongfen Tong

**Affiliations:** School of Environmental and Chemical Engineering, Nanchang Hangkong University, 696 Fenghe South Avenue, Nanchang 330063, China

**Keywords:** gel polymer electrolytes, chitosan, self-healing, ionic liquid, supercapacitor

## Abstract

Gel polymer electrolytes with a satisfied ionic conductivity have attracted interest in flexible energy storage technologies, such as supercapacitors and rechargeable batteries. However, the poor mechanical strength inhibits its widespread application. One of the most significant ways to avoid the drawbacks of the gel polymer electrolytes without compromising their ion transportation capabilities is to create a self−healing structure with the cross−linking segment. Herein, a new kind of macromolecule chemical cross−linked network ionic gel polymer electrolyte (MCIGPE) with superior electrochemical characteristics, a high flexibility, and an excellent self−healing ability were designed, based on chitosan and dibenzaldehyde−terminated poly (ethylene glycol) (PEGDA) via dynamic imine bonds. The ionic conductivity of the MCIGPE−65 can achieve 2.75 × 10^−2^ S cm^−1^. A symmetric all−solid−state supercapacitor employing carbon cloth as current collectors, activated a carbon film as electrodes, and MCIGPE−65 as a gel polymer electrolyte exhibits a high specific capacitance of 51.1 F g^−1^ at 1 A g^−1^, and the energy density of 7.1 Wh kg^−1^ at a power density of 500.2 W kg^−1^. This research proves the enormous potential of incorporating, environmentally and economically, chitosan into gel polymer electrolytes for supercapacitors.

## 1. Introduction

With the incredible market expansion of flexible and wearable electronic devices, it is vital to examine the development of lightweight, flexible, and safe energy storage devices [1,2]. Because of their quick charge/discharge rates, extended cycle life, and high power density, flexible capacitors are regarded as prospective high−performance energy storage technologies that have attracted significant research attention [3]. Usually, liquid electrolytes are used to fabricate the electrochemical double−layer capacitors (EDLCs), and the disadvantages, such as leakage and corrosion, will seriously reduce safety levels [4]. Solid polymer electrolytes solve the problems of liquid leakage and harsh assembly environment. Still, the low ionic conductivity and high contact resistance between electrolyte and electrode materials limit their further application [5,6]. Gel polymer electrolytes fusing the advantages of liquid and solid electrolytes with flexibility, good processability, and excellent electrochemical properties, have attracted wide attention and research opportunities [7,8].

Biopolymer−based electrolytes with environmentally friendly properties are commonly used in electrical energy storage devices, such as EDLC and batteries [9,10]. Chitosan (CS) is a potential natural bio−polymer generated from chitin that is a plentiful, renewable, biodegradable, cost−effective, and non−toxic biodegradable polymeric material [11]. Due to the fact that CS possesses various functional groups, such as amine (NH_2_), hydroxyl (OH) groups, and glycosidic linkages (C−O−C), these cause a chemical reaction with different inorganic salts and supply free mobile ions to the system [12,13]. Kotatha et al. studied a novel ionic liquid gel polymer electrolyte, based on chitosan with ionic liquids (EMImBF_4_), which showed a high thermal stability and high ionic conductivity [14]. Alves et al. investigated a system comprised of a chitosan host polymer, samarium triflate (Sm (CF_3_SO_3_)_3_), and glycerol. The prepared electrolytes can achieve the maximum conductivity values of 3.65 × 10^−7^ S cm^−1^ at 30 °C [15]. Aziz et al. also reported a solid polymer electrolyte (SPE), based on chitosan, methylcellulose, and NH_4_I with the highest conducting of 6.65 × 10^−4^ S cm^−1^ [16].

Additionally, flexible capacitors will inevitably be damaged in practical application. Therefore, composite gel polymer electrolytes with a self−healing ability have attracted much attention in developing portable and wearable electronics [17,18]. The self−healing polymer electrolytes that have been reported most rely on supramolecular interactions, such as hydrogen−bonding, electrostatic interaction, host−guest recognition, metal−ligand coordination, hydrophobic association, π−π stacking, or dynamic covalent bonds [19,20,21]. The Schiff base linkage (−HC=N−) is a type of dynamic covalent connection formed by aldehyde and amino groups (−NH_2_) [22]. Although plenty of dynamic polymer electrolytes have been reported, the most challenging issue that needs to be solved in the polymer electrolytes with a self−healing ability is to explore the materials with an excellent healing efficiency without any external stimulation.

An innovative self−healing macromolecule chemical cross−linked network ionic gel polymer electrolyte (MCIGPE), based on chitosan and the ionic liquid, was prepared for a flexible supercapacitor in this paper. Dibenzaldehyde−terminated poly (ethylene glycol) (PEGDA) and chitosan were cross−linked by the Schiff base reaction to generate a 3D network polymer structure. EMImBF_4_ with a higher thermal stability and LiBF_4_ with a lower lattice energy were selected as ionic liquid (IL) electrolytes. The hydrogen bonding between the hydroxyl groups in chitosan and the highly reversible imine bond in the cross−linking point endow MCIGPE with a rapid self−repairing ability to heal mechanical damage at room temperature without external stimulation. The flexible sandwiching supercapacitors were assembled with MCIGPE as an electrolyte and activated carbon film as an electrode material. The capacitance, and deformation capability of the device were also studied. The MCIGPEs maintain not only a good flexibility and excellent self−healing ability, but also possess a superior electrochemical performance, which provides a potential application for energy storage devices.

## 2. Results and Discussion

### 2.1. Structural Characterization of Polymers and Electrolytes

**Figure 1:**
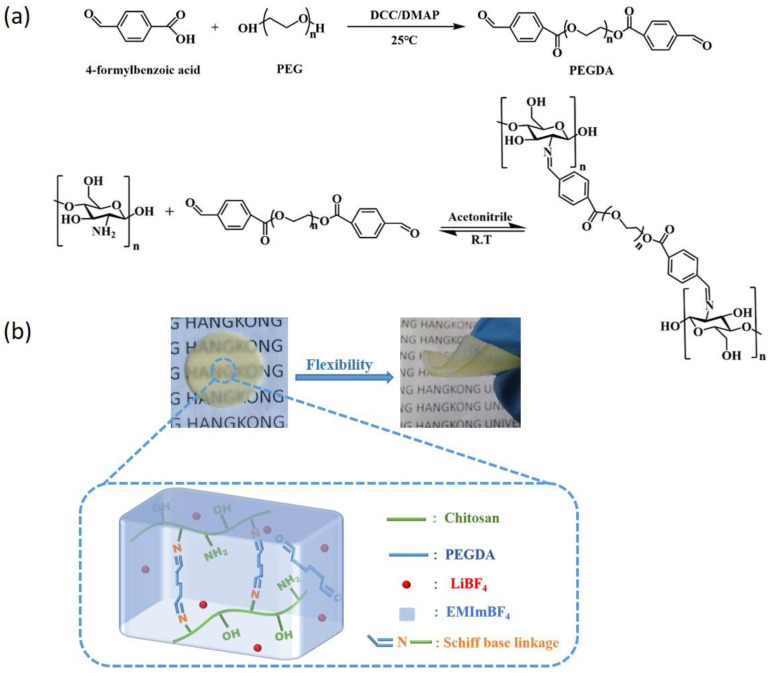
(**a**) Synthetic route of PEGDA and the cross−linked polymer, (**b**) schematic illustration and digital photos of the cross−linked MCIGPE film.

The macromolecule based on chitosan, was completely cross−linked to generate an interconnected bulk through the interplay of the Schiff base −HC=N− and hydrogen bonds. The synthetic route for the dibenzaldehyde−terminated poly (ethylene glycol) (PEGDA) and cross−linked macromolecular polymer were illustrated in Figure 1a. The cross−linked network ionic gel polymer electrolyte (MCIGPE) was prepared with the addition of 0.1 mol kg^−1^ LiBF_4_/EMImBF_4_ in the cross−linked polymers. The gel polymer electrolytes were designated by the weight percentage of their components, and referred to as MCIGPE−0, MCIGPE−20, MCIGPE−35, MCIGPE−50, MCIGPE−65. For example, MCIGPE−20 represents a sample with 20 wt.% LiBF_4_/EMImBF_4_ (0.1 mol kg^−1^) and 80 wt.% cross−linked polymers. At the same time, dynamic covalent imine bonds and hydrogen bonds are introduced into the macromolecule structure as reversible self−healing sites, and the ionic liquids are in charge of enhancing the electrochemical performance and stability. (Figure 1b) It can be seen from the digital photos that the MCIGPE is transparent and bendable, implying that it is suitable to apply in flexible electrochemical devices.

**Figure 2:**
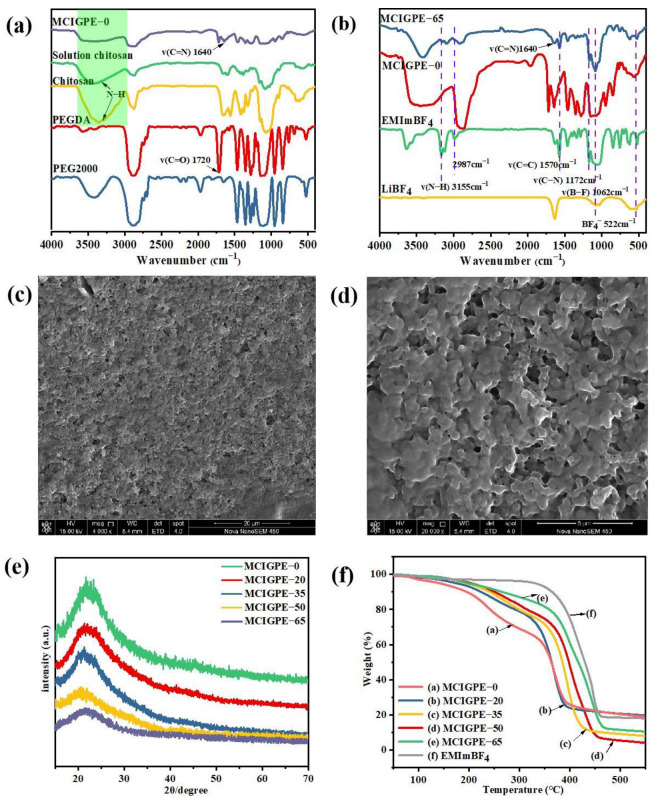
(**a**) FT−IR spectrum of PEG2000, PEGDA, solution chitosan, chitosan and MCIGPE−0; (**b**) LiBF_4_, EMImBF_4,_ MCIGPE−0 and MCIGPE−65 in the range of 4000~400 cm^−1^; SEM of MCIGPE−0 at (**c**) 20 μm and (**d**) 5 μm magnifications; (**e**) X−ray diffraction (XRD) of MCIGPEs contain different IL; (**f**) TGA curves of MCIGPE with different ionic liquids in a nitrogen atmosphere.

The structures of the medium and composite electrolytes are confirmed by FT−IR spectra. As shown in Figure 2a, the stretching vibration of the C=O bond in the aldehyde group contributed to the new peak of 1720 cm^−1^ in PEGDA, confirming that −CHO was effectively grafted to PEG2000 [23]. The stretching vibration of chitosan −OH and −NH_2_ groups can be linked to the wide broad adsorption peak from 3700 cm^−1^ to 3000 cm^−1^ (shown with green shadow). Following the cross−linking with PEGDA, the adsorption peak of the −NH_2_ groups was reduced, demonstrating the consumption of the −NH_2_ groups. The disappearing height for aldehyde at 1720 cm^−1^ in PEGDA and the existence of a new mount at 1640 cm^−1^ related to the C=N stretching vibration in the spectrum of MCIGPE−0, manifesting that the MCIGPE−0 polymer was successfully formed via the Schiff base reaction [24]. The FT−IR spectra of EMImBF_4_, LIBF_4_, MCIGPE−0, and MCIGPE−65 were also compared in Figure 2b. The new peaks at 3155 cm^−1^ and 2987 cm^−1^ were caused by the imidazolium ring’s aliphatic asymmetric stretching vibrations, and the peaks corresponding to the C−H tensile changed to a relative lower site, demonstrating the interaction between the polymer and ionic liquid. The double bond and C–N stretching vibration of the imidazolium structure is responsible for the strong peak at 1570 cm^−1^ and 1172 cm^−1^. In particular, the ridge at 1062 cm^−1^ and 522 cm^−1^ corresponds to the beats of the BF_4_^−^ anion in LiBF_4_ [14,25]. The FT−IR spectra demonstrate that EMImBF_4_ was successfully incorporated into the MCIGPE films.

### 2.2. Morphology and Thermal Behaviors of the Electrolytes

A scanning electron microscope (SEM) was used to examine the surface morphology of the MCIGPE−0 polymer. As shown in Figure 2c,d, the MCIGPE−0 possesses a network of macrospores that give enough area for absorbing the ionic liquid and aid in the passage of conductive ions in the polymer electrolyte matrix [26]. The porous structure may be derived from the cross−linking domains between the polymer chains and the solvent evaporation [14].

The crystallinity of a polymer electrolyte membrane is an important parameter to measure its ion transport capacity. The XRD patterns of the polymer MCIGPE−0 and electrolyte MCIGPE 20~65 were shown in Figure 2e. It can be seen that there are apparent peaks at 21.3° and 23.7° for the polymer MCIGPE−0 without ionic liquid, indicating that it is a semi−crystalline structure. For MCIGPE−20~65, the intensities of the XRD peaks decrease with the increasing ionic liquid content, verifying the formation of the amorphous state. It is due to the polymer−LiBF_4_ salt complexation, which results in a reduction in the intermolecular or intramolecular contacts. Simultaneously, the appropriate EMImBF_4_ plasticizer in the MCIGPEs matrix can effectively destroy the hydrogen bond force in the chitosan matrix, thus enhancing the disorder of the chitosan molecular structure, finally leading to the decrease of crystallinity for the electrolyte membrane [27]. Furthermore, no extra peaks emerge in the MCIGPEs film, proving that the LiBF_4_ and EMImBF_4_ plasticizers have been completely dissolved in the polymer matrix.

The TGA curves of EMImBF_4_, MCIGPE−0, MCIGPE−20, MCIGPE−35, MCIGPE−50, and MCIGPE−65 are shown in Figure 2f. A weak weight loss at approximately 100 °C may be induced by the evaporation of the volatile content and liquid evaporation. The thermogravimetric curves of MCIGPE−20, MCIGPE−35, MCIGPE−50, and MCIGPE−65 all have two evident weight loss processes. The first stage was found at around 250 °C, and the weight loss was about 17% to 23%, ascribed to the chitosan decomposition. It was reported that chitosan delivers the biochemical degradation and deacetylation near 275 °C [28]. The second−stage degradation temperatures of MCIGPE−20, MCIGPE−35, MCIGPE−50, and MCIGPE−65 were 324 °C, 349 °C, 364 °C, and 373 °C, respectively, which were mainly attributed to the decomposition of EMImBF_4_ and the primary chain fracture of the polymer matrix [14,29]. The degradation temperature increased as the ionic liquid content (EMImBF_4_) of the electrolyte increased, implying that EMImBF_4_ was incorporated into the MCIGPEs electrolyte successfully. Therefore, the gel polymer electrolytes have a potential application in EDLCs at high temperatures.

### 2.3. Self−Healing Property

**Figure 3:**
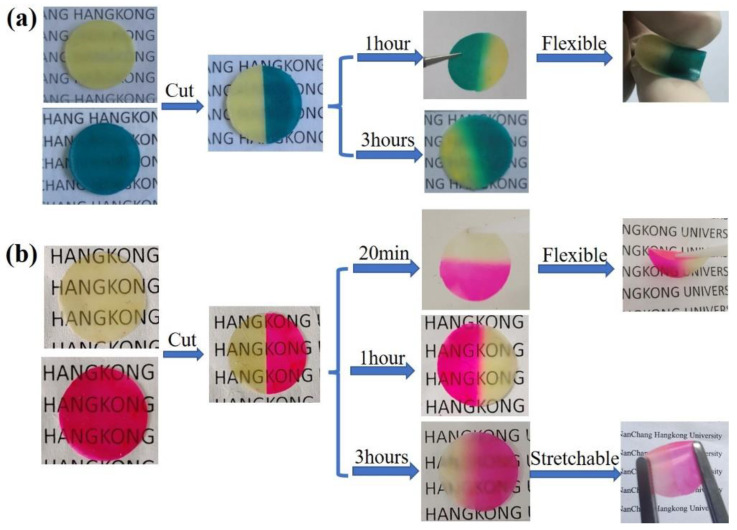
Self−healing process of (**a**) polymer MCIGPE−0 and (**b**) gel polymer electrolyte MCIGPE−65 at room temperature.

The self−healing polymer is an ideal material due to its ability to prolong lifetime and lower replacement costs in practical applications [30]. A digital camera was used to record the self−healing process of MCIGPE−0 and MCIGPE−65. (Figure 3). A fragment of the MCIGPE−0 film was colored with an indicator (rhodamine B) to enable a more intuitive observation. The polymer, either with or without rhodamine B, was sliced into two parts. The two semicircular polymer films with different colors were quickly assembled between fresh cuts. The breakage was healed within 1 h at room temperature without external stimulation, and the incision interface turned green, indicating that a small amount of dye was spreading through the healed interface. Then, 3 h later, the incision was recovered to its original entirety, and the repaired sample maintained an excellent flexibility (Figure 3a). The ideal self−healing property of the cross−linked polymer is attributed to the dynamic imine metathesis and supramolecular interaction, based on the hydrogen bond [31]. To compare the self−healing performance of the ionic gel electrolytes, the recoverability of MCIGPE−65 was also tested at room temperature (Figure 3b). The gel interface was reattached within 20 min, and the repaired one could be bent without rupture. The fast self−healing process of the gel electrolytes indicates that ionic liquids are beneficial in enhancing the repair efficiency. Then, 3 h later, it was observed that the interface entirely disappeared and the electrolyte could be stretched without breaking. The diffusion range was more extensive than the MCIGPE−0, implying that ionic liquids can promote the ion transport and molecular movement [32]. In general, the applicability of gel electrolytes in capacitors is expanded by introducing ionic liquids, which provide a superior self−healing ability.

### 2.4. Electrochemical Properties of the Electrolytes

**Figure 4:**
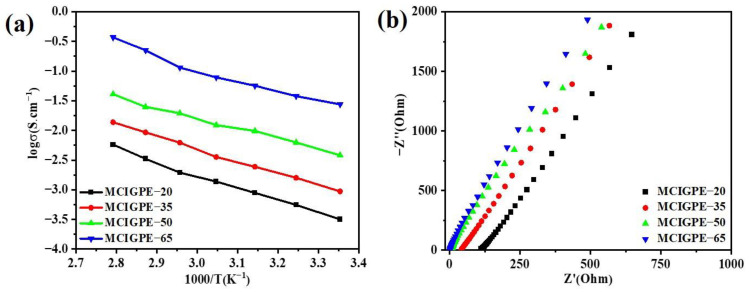
(**a**) The ionic conductivity of the ionic gel polymer electrolytes containing 20~65% ionic liquids in the temperature range from 25 °C to 85 °C, (**b**) impedance spectra of the ionic gel polymer electrolytes at 35 °C.

The ionic conductivity is a fundamental metric to represent the ionic transport capacity for electrolytes. Figure 4a presents the ionic conductivity of different ionic liquid−loaded MCIGPEs from 25 to 85 °C. The ionic conductivity of the electrolyte rises with the increasing temperature for all samples, and the ionic conductivity value similarly rises with an increase in the ionic liquid content. According to Equation (1), the conductivity value of MCIGPE−20, MCIGPE−35, MCIGPE−50 and MCIGPE−65 is 3.17 × 10^−4^, 9.38 × 10^−4^, 3.80 × 10^−3^, and 2.75 × 10^−2^ S cm^−1^, respectively. The excellent electrochemistry ability is because the porous cross−linked polymer aids in the locking of more liquid electrolytes, which promotes the ion transportation and mobility. The highest ionic conductivity value of MCIGPE−65 should also be ascribed to the reduced crystallization of the polymers, which is consistent with the results of the XRD. Figure 4b depicts the typical impedance diagrams of MCIGPEs with various ionic liquids at 35 °C. It can be demonstrated that all MCIGPEs have a single slant curve in the high−frequency region of the Nyquist diagram.

**Figure 5:**
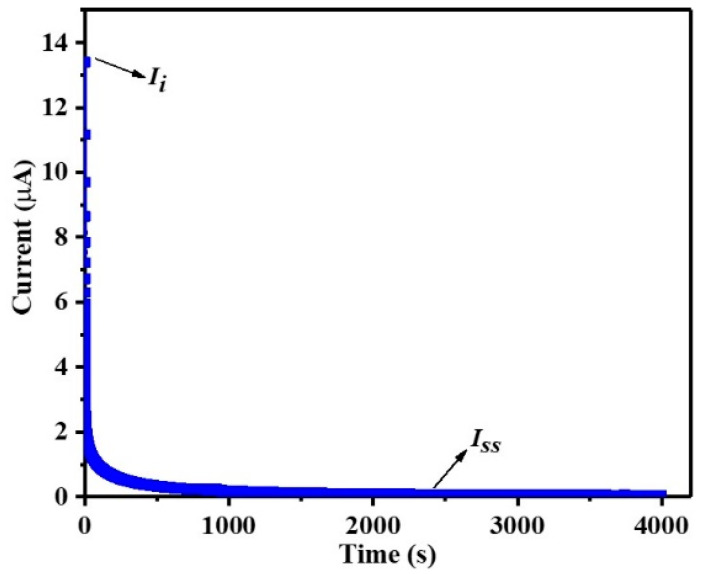
The polarization plot of the current versus the time of the MCIGPE−65.

Figure 5 presents the polarization plot of the current versus the time for MCIGPE−65. The MCIGPE−65 electrolyte film was placed in the middle of the symmetrical stainless steel (SS) electrode, and a constant voltage of 0.5 V was used, at room temperature. The continuous current value presents the electrolyte’s ionic conductor behavior, where *I_ss_* and *I_i_* are calculated to be at 0.12 and 13.39, respectively. The relatively high initial current is caused by the participation of the ions and electrons in conduction, and the stainless steel has a strong ionic−blocking effect [33]. As time goes on, the current rapidly drops and remains almost flat. Using Equations (2) and (3), the values of *t_ion_* and *t_el_* for the electrolyte can be calculated as 0.99 and 0.01, respectively. The current flowing through the electrode of an ionic conductor decreases fast with time, but the current of a non−ionic conductor does not diminish with time [11]. The result indicates that the ions dominate the prepared ionic gel electrolyte.

### 2.5. EDLC Cells Performance

#### 2.5.1. Cyclic Voltammetry and Galvanostatic Charge−Discharge Analysis

**Figure 6:**
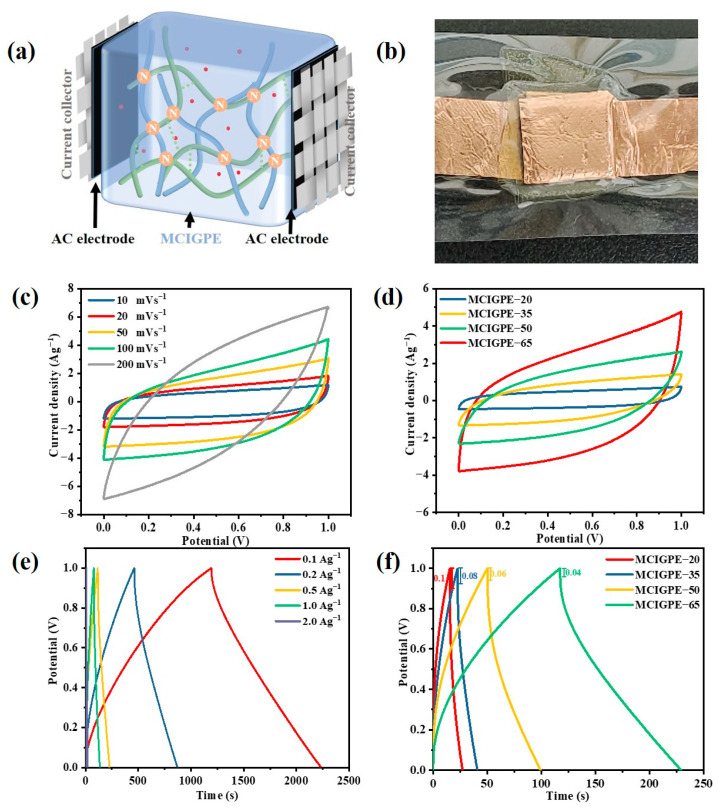
(**a**) Schematic diagram of the solid state EDLC, (**b**) Digital photos of the assembled EDLC device, (**c**) CV curves of the EDLC, based on MCIGPE−65 at various scanning rates, (**d**) CV curves of the EDLC, based on MCIGPEs at 100 mV s^−1^ scan rates. (**e**) The galvanostatic charge−discharge (GCD) curves of the EDLC, based on MCIGPE−65 at different scanning rates and (**f**) MCIGPEs with a different IL content at 0.5 A g^−1^.

The MCIGPEs films provide a high ionic conductivity and excellent flexibility, which can be satisfied in the EDLC. The schematic design of the constructed flexible EDLC (Figure 6a,b) shows three layers: activated carbon film, MCIGPEs membrane, and activated carbon film. The MCIGPE membrane acted as an electrolyte and separator between the two symmetrical activated carbon electrodes, then attached the conductive tape to carbon cloth, finally, the cell was packaged and heat sealed at 90 °C.

The CV curves of MCIGPE−65 with a potential window of 0~1 V for the EDLC at 10~200 mV s^−1^ scan rates, were shown in Figure 6c. When the scan rate increased from 10 mV s^−1^ to 200 mV s^−1^, the diagram turned from a nearly quasi−rectangular shape to a leaf shape. It is mainly due to the internal resistance and carbon porosity, which can affect the current, eventually showing different shapes [34]. Moreover, the ions’ conduction is fast at a high scan rate, however, the ions have sufficient time to diffuse to all vacancies in the active electrode material at low scan rates [11]. The absence of redox peaks in the CV plots indicates that the capacitance is retained via an ion accumulation between the electrode−electrolyte interfaces, which exhibit double−layer capacitance features [35].

Figure 6d shows the CV curves of the EDLC, based on MCIGPE−(20~65) with a potential window of 0~1 V at 100 mV s^−1^ scan rates at room temperature. The integrated area of the CV curves grew dramatically from MCIGPE−20 to MCIGPE−65, as the amount of ionic liquid in the electrolyte increased. For MCIGPE−65 with the highest ionic liquid content, the corresponding curve remains similar to a symmetrical and rectangular shape, indicating the relative ideal capacitive behavior and lower charge transfer resistance, due to the high ionic conductivity [36]. The excellent electrochemistry performance ensures that the MCIGPEs electrolyte membrane is more suitable for the application in the EDLC.

The galvanostatic charge−discharge (GCD) technique was also helpful for assessing the capacitive characteristics. Figure 6e presents the charging and discharging performance of the EDLC, based on MCIGPE−65 at a current density of 0.1~2 A g^−1^ between 0 to 1 V. At the interface between the gel electrolytes and the AC electrode, the reversible charge and discharge is shown by the symmetrical charge−discharge curves. According to Equation (4), the specific capacitances of the device were 104.1, 81.4, 57.5, 51.1, and 21.2 F g^−1^ at a current density from 0.1 to 2 A g^−1^, respectively. The reduction in the ion diffusion into the double−layer electrode and the rise in the material electrical resistance caused the specific capacitances to decrease with an increase in the current density. The power and energy density were critical indices for assessing the supercapacitor electrochemical characteristics. According to Equations (5) and (6), the energy density of the device reached 7.99 Wh kg^−1^ at a power density of 250.12 W kg^−1^, and remained 2.94 Wh kg^−1^ at a power density of 999.84 W kg^−1^ at 2 A g^−1^. Additionally, the voltage drop is hardly noticeable at low current densities. As the current density grows, it becomes more apparent because fewer ions are diffused into the double−layer electrode, and the resistance rises [36].

Figure 6f shows the GCD curve of MCIGPE, based on the EDLC with a different IL content at the current density of 0.5 A g^−1^. It can be seen that each turn has an approximately symmetrical isosceles triangle, indicating that the charge and discharge reversibility of the supercapacitor is excellent. The characteristics of the double layer capacitance are the same as the CV curve (Figure 6d). All of the curves were symmetric triangular in shape, with a minor internal resistance decrease. The presence of the resistance between the current collector and the electrolyte, causes the IR to decrease during the discharge [37]. The MCIGPE−65 GCD curve has a relatively symmetric triangular shape with a minimum internal resistance drop of 0.04 V, demonstrating the double layer capacitor’s optimum reversible capacitance feature. Table 1 summarized the performance of several EDLCs with chitosan−based electrolytes, which shows that the performance of the EDLC with MCIGPEs as electrolytes, is comparable to the reported ones.

#### 2.5.2. Cycle−Stability Analysis

**Figure 7:**
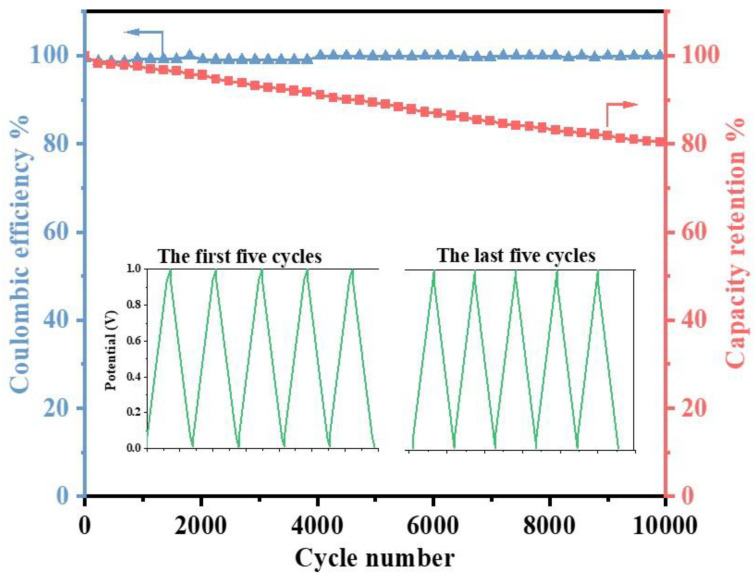
Cycling performance of the EDLC with MCIGPE−65 at a current density of 1 A g^−1^. (Inset: GCD curve of the first five cycles and last five cycles).

Another important quality for the actual application of supercapacitors is the cycle stability. For the EDLC using the MCIGPE−65 electrolyte, the test was performed for 10,000 cycles at a current density of 1 A g^−1^ (Figure 7). The capacitance can still maintain 80.4%. The Columbic efficiency remains about 99.7% after the 10,000 charging−discharging cycling, indicating that the manufactured supercapacitor with MCIGPE−65 as an electrolyte possesses a high degree of reversibility and a strong electrochemical stability.

### 2.6. Electrochemical Performance of Flexible Supercapacitors

**Figure 8:**
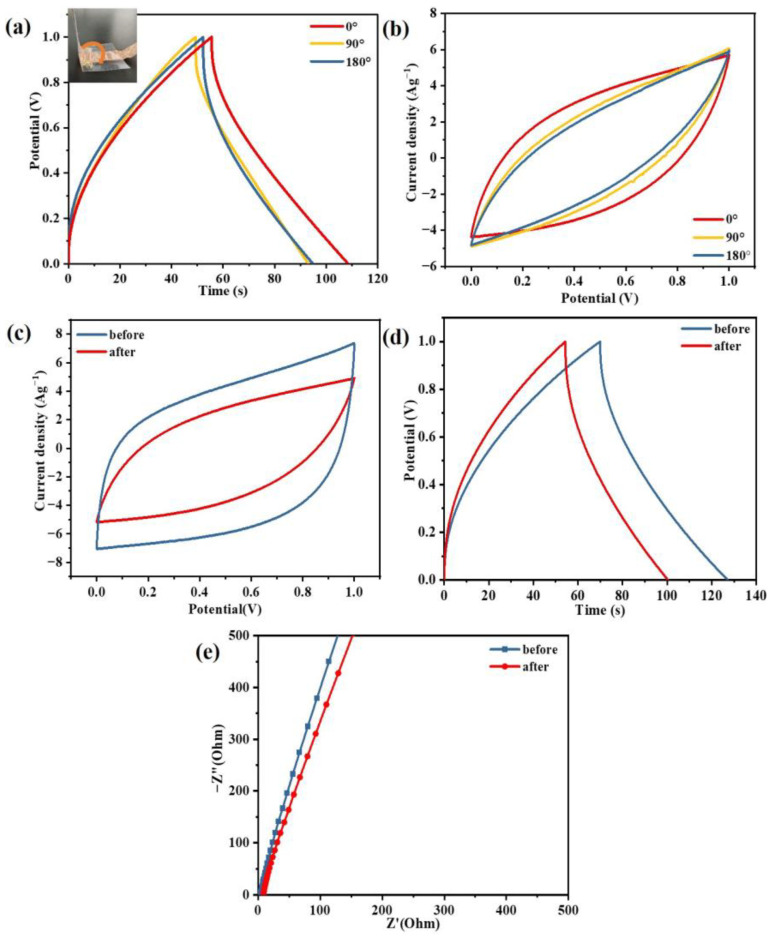
(**a**) CV curves at a scan rate of 100 mV s^−1^ for the EDLCs, based on MCIGPE−65 with different bending angles (the inset show the digital photo of the EDLC bend to 90°), (**b**) GCD profiles at a current density of 1 A g^−1^, (**c**) CV curves at 100 mV s^−1^, (**d**) GCD profile at 1 A g^−1^, and (**e**) impedance spectra of MCIGPE−65 at 35 °C before and after the healing cycle.

Given the excellent cohesiveness of the gel electrolytes with the electrode, the assembled EDLC can be easily bent at different angles without peeling. The bent EDLC also exhibits a good CV curve, even at 180° (Figure 8a). It still exhibited triangular GCD profiles with a very slight deviation at different bending angles (Figure 8b). The electrode−specific capacitances from 0° to 180° are obtained as 52.6, 43.7, and 42.5 F g^−1^, respectively, and all the capacitance retention rates remain around 83%. A little drop in capacitance is ascribed to the weaker adhesion between the MCIGPE membrane and the activated carbon electrode, due to the bending [42]. The remarkably stable capacitive performance under various bending states proves the device possesses an excellent flexibility and mechanical toughness. It can be considered that the cross−linked ionic gel electrolytes, based on chitosan, are a promising candidate for the construction of flexible supercapacitors with a superior performance.

To further confirm the self−healing ability of the electrolytes, the capacitance characteristics of the EDLCs with MCIGPE−65 were tested before and after cutting/self−healing. At a scan rate of 100 mV s^−1^, the CV curves of the capacitor were measured before and after healing. (Figure 8c), the GCD test was evaluated at a current density of 1 A g^−1^ (Figure 8d), and the EIS test with stainless steel electrodes in Figure 8e. It was found that the CV and GCD tests showed a slight decrease after cutting and self−healing. The decline may be due to the activated carbon being introduced into the gel electrolyte interface during the cutting process, and the self−healing ability of the electrolytes being hindered by the impurities [43]. The EIS values of the electrolytes have also been tested before and after self−healing. According to the calculation of Equation (1), the electrical conductivity is 2.31 × 10^−2^ S cm^−1^, and remaining 2.06 × 10^−2^ S cm^−1^ after the repair, replying that the cut−off repair has little impact on the electrochemistry of the ionic gel electrolytes. Although the capacitance decreases after healing, MCIGPE−65, with its self−repairing ability, can avoid mechanical failure and retain the device’s integrity.

**Figure 9:**
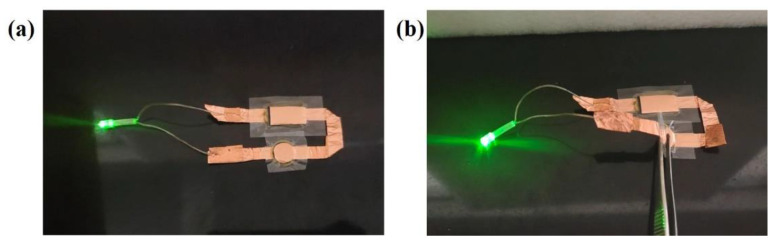
Digital photo of a LED activated by the supercapacitor under (**a**) an unfolded condition and (**b**) a bent state at 90°.

The soft package capacitor was designed and tested to examine the flexibility and possible applications of the gel polymer electrolyte membranes. As shown in Figure 9, the two capacitors were connected with a series of conductive strips, and the light−emitting diode (LED) was fixed on both sides of the capacitor. Then, the pouch cell assembled with MCIGPE−65 can successfully light up the green LED lamp under unfolded conditions and a 90° bend state at room temperature. This phenomenon further proved that chitosan−based gel polymer electrolyte membranes could be successfully used in practical applications.

## 3. Conclusions

A novel ionic gel polymer electrolyte, based on cross−linking chitosan and ionic liquid with dynamic Schiff and hydrogen bonding interactions, were prepared in this study. The high conductivity and amorphous shape of the electrolyte are due to the excellent interaction between the polymer segment and the ionic liquid. Without external stimulation, the MCIGPEs have an exceptional flexibility and outstanding self−healing characteristics with a (*t_ion_*) value of 0.99. The TNM analysis indicated that the ions are the major charge carrier. The electrochemical capacity of a the symmetric quasi−solid−state EDLC integrating the MCIGPE−65 film and activated carbon electrode show a capacitance of 51.1 F g^−1^ with an energy density of 7.1 W h kg^−1^, a power density of 500.2 W kg^−1^ at 1 A g^−1^ and a suitable device cyclic stability with a capacitance retention above 80.4% after 10,000 cycles. Therefore, the ionic gel polymer electrolyte with a self−healing ability offers a potential and innovative application to develop flexible energy storage devices.

## 4. Material and Methods

### 4.1. Materials

Chitosan (deacetylation rate >95%) was purchased from Shanghai Bide Medical Science and Technology Co., Ltd., Shanghai, China. Poly (ethylene glycol) with Mn of 2000 (PEG2000), 4−formylbenzoic acid, 4−(dimethylamino) pyridine (DMAP) and N,N’−dicyclohexylcarbodiimide (DCC) were all purchased from Saen Chemical Technology Co., Ltd., Shanghai, China. The hydrogen peroxide solution (H_2_O_2_, 30 wt%), lithium hydroxide (LiOH), acetonitrile (AR), tetrahydrofuran (THF), acetic acid and ethyl alcohol were provided by Sinopharm Co., Ltd., Shanghai, China. 1−ethyl−3−methylimidazolium tetrafluoroborate (EMImBF_4_, 99.0%) and lithium tetrafluoroborate (LiBF_4_) were purchased from Sahn Chemical Technology Co., LTD., Shanghai, China.. Activated carbon (AC), polyvinylidene fluoride (PVDF) and conductive carbon black were purchased from Canrd New Energy Technology Co., Ltd. Guangdong, China. All other chemicals were of analytical grade and used without further purification.

### 4.2. Preparation of the Soluble Chitosan

H_2_O_2_ triggered a breakdown process that produced a soluble chitosan powder [44]. Typically, 100 mL 1 wt% acetic acid water solution was added to 1 g chitosan powder to create a transparent and viscous solution. The chitosan solution was then dropped−wise infused with 20 mL of a 0.88 ml/L H_2_O_2_ solution, while being magnetically stirred. For 12 h, the solution was allowed to deteriorate at 60 °C. A diluted solution was produced after the degrading process. Once the solution was neutralized with a 7 wt% LiOH solution, the insoluble chitosan was sorted out. The phase transformation was used to collect the product, which was subsequently freeze−dried and vacuum−dried at 60 °C for 24 h.

### 4.3. Synthesis of Dibenzaldehyde−Terminated Poly (Ethylene Glycol) (PEGDA)

PEGDA was synthesized, according to [23,45]. In 150 mL of dry THF with N_2_ protection, PEG2000 (4.89 g), 4−formylbenzoic acid (1.47 g), and DMAP (0.075 g) were dissolved. The solution was then supplemented with DCC (2.52 g) and reacted at room temperature for 18 h. The reaction solution was centrifuged and the supernatant was precipitated with diethyl ether to obtain a white solid. Then, the solid precipitated in the diethyl ether was re−dissolved in THF and centrifuged again, the process of dissolution and precipitation was repeated three times, to improve the purity of the product. The finished product was dried at room temperature in a vacuum oven.

### 4.4. Preparation of MCIGPE

To prepare the MCIGPE membranes, the soluble chitosan powder was dissolved in acetonitrile before adding PEGDA. To ensure the high functional group conversion, the initial concentrations of the functional groups ([amine]_0_ and [aldehyde]_0_) are maintained at 0.2 M. The weight ratio of the cross−linked polymers was used to calculate the quantity of 0.1 mol kg^−1^ LiBF_4_/EMImBF_4_. They were transported to volatile liquids in PTFE abrasives after evenly mixing and stirring at room temperature, and the MCIGPEs membrane was formed by gelation. The gel polymer electrolytes were labeled MCIGPE−20, MCIGPE−35, MCIGPE−50, and MCIGPE−65, based on the weight proportion of their components. For example, MCIGPE−20 is a sample with 20 wt.% LiBF_4_/EMImBF_4_ (0.1 mol kg^−1^) and 80 wt.% cross−linked polymers. The thickness of the gel polymer film is maintained at about 120–150 μm.

### 4.5. Preparation of the Activated Carbon Electrode

The active electrode was made, as follows [46]: activated carbon (80 wt.%), acetylene black (10 wt.%) and polyvinylidene fluoride (PVDF) (10 wt.%).The PVDF was dissolved in N−methyl−2−pyrrolidone (NMP). The activated carbon, acetylene black, and NMP−PVDF solution were ground on a planetary ball miller for 20 min at the speed of 500 r min^−1^, to obtain a thick black solution. Using a coating machine, we applied this activated carbon solution to the carbon fabric. The electrode was dried in a 60 °C oven. The area cut from the prepared activated carbon film is a 1.2 × 1.2 cm^2^ small block for the subsequent assembly of the capacitors. Each block was loaded with 2 mg of activated carbon. The activated carbon electrode in the flexible device is a rectangle with a diameter of 1.2 × 2.4 cm^2^.

### 4.6. Assembly of the All−Solid−State EDLC

The symmetric activated carbon film was employed as the electrode, the current collector was made of carbon cloth, and the electrolyte was made of MCIGPE film. The MCIGPEs membrane was placed between two sheets of activated carbon (Figure 6a).

### 4.7. Structural Characterization of IGPE

Infrared spectroscopy was carried out in the 4000–400 cm^−1^ on the Shimadzu IR Prestige−21 Fourier transform infrared spectrophotometer (FT−IR). We analyzed the MCIGPEs’ composition and structure. The electrolyte films were directly examined using a membrane test mold, while the powder samples were analyzed using the potassium bromide tablet method. The Bruker D8 Focus X−ray diffractometer was used to test the XRD. A silicon glass sheet is bonded to an electrode film sample with a flat surface, and the sample is examined at room temperature. The test scan angles vary from 0° to 70° with a scan rate of 1° min^−1^. A scanning electron microscope (ESEM; FEI Quanta 200) was used to examine the membrane’s morphology. The conductive polymer film can be directly glued to the conductive adhesive, and the non−conductive polymer film can be sprayed with gold.

### 4.8. Thermal Analysis

The thermodynamic properties of MCIGPEs were analyzed by a thermogravimetric analysis (TGA). The rate is characterized in the range of 50 °C to 600 °C and use a TA Q20 analyzer to heat at 10 °C min^−1^ under N_2_ atmosphere.

### 4.9. Self−Healing Properties

We cut the stained IGPE into two parts using a razor, and used tweezers to reattach the two halves of the membrane at the cutting interface for a certain amount of time. We monitored the rate of healing at different temperatures. We collected the macro photos with a camera.

### 4.10. Electrochemical Measurement

In this paper, the electrochemical properties of MCIGPE include an AC impedance test (EIS) and a transference number measurement (TNM) analysis was carried out using a Chenhua CHI760 electrochemical workstation.

The ionic conductivity of the MCIGPEs can be obtained by the AC impedance spectroscopy (EIS) with a blocking battery, which is assembled in stainless steel (SS)/electrolyte/stainless steel (SS) and fixed in a mold, and the test is repeated three times for each sample. The frequency range is from 1 Hz to 100 KHz, the amplitude is 5 mV, and the temperature range is from 25 °C to 85 °C. Prior to testing, we left the blocked battery at each temperature for 1 h, to attain the thermal equilibrium. The following equation is used to calculate the ionic conductivity of an electrolyte:

(1) $$\sigma =d/s\times R_{b}$$

where *d* indicates the thickness of the MCIGPE film, *s* indicates the contact area between the MCIGPE films and the electrolyte, *R_b_* is the bulk resistance determined from the first intercept on the impedance spectrum’s x−axis.

The transference number measurement (TNM) study was performed under DC conditions by applying a constant voltage at both ends of the battery module. The operating voltage for this analysis was 0.5 V where the cell was polarized, versus the time at room temperature. The transference number of ions (*t_ion_*) and electrons (*t_el_*) can be obtained from the following equation:

(2) $$t_{ion}=\frac{I_{i}-I_{ss}}{I_{i}}$$

(3) $$t_{el}=1-t_{ion}$$

Here the initial and steady−state current are denoted as *I_i_* and *I_ss_*, respectively.

### 4.11. EDLC Measurement

The measurements of the cyclic voltammetry (CV) and galvanostatic charging−discharging (GCD) of the flexible supercapacitor were conducted on an electrochemical workstation CHI660E (Shanghai, China). The double−layer capacitor scans at 10~200 mV s^−1^ sweep speed in the 0~1 V voltage range, or at a constant sweep speed in the 0–1 voltage range. According to the shape of the cyclic voltammetry curve, we evaluated and analyzed the performance of the double−layer capacitor.

The GCD was used to test the charge−discharge performance of the supercapacitor at different current densities (0.1, 0.2, 0.5, 1, 2A g^−1^). The specific capacitance (*C*, F g^−1^), the energy (*E*, Wh kg^−1^), and the power density (*P*, W kg^−1^) can be calculated by Formulas (4)–(6) [38].

(4) C=I×Δt/(ΔV×m)

(5) $$E=C\times V^{2}/\left(2\times 3.6\right)$$

(6) P=E×3600/Δt

where *I* (A) was voltametric discharging current, Δ*t* (s) was the discharging time, Δ*V*(V) was the potential window, and *m* (g) was the mass of active materials.

The GCD cycling was examined by a battery test equipment (CT−4008Tn−5V10mA−164, NEWEAR Technology Co, Ltd., Shenzhen, China) with current densities of 1A g^−1^ at a potential range of 0~1 V.

## Figures and Tables

**Table 1:** Chitosan−based EDLCs reported by other works.

Membrane	Electrodes	σ (mS/cm)	C (F/g)	E (Wh/kg)	P(W/kg)	Ref.
Carboxylated chitosan/HCI	AC	8.69 × 10^−2^	45.9 at 0.5 Ag^−1^	5.2	226.6	[38]
Chitosan/cellulose/EMIMAc	AC	2.1	33.1 at 10 mVs^−1^	/	/	[39]
Chitosan/dextran/LiClO _4_	AC	5.16 × 10^−3^	8.7 at 0.5 mA cm^−2^	1.21	685	[40]
Chitosan/PEO/LiClO_4_	AC	7.34 × 10^−4^	6.88 at 0.5 mA cm^−2^	0.94	305	[41]
Chitosan/Mg (CH_3_COO)_2_/glycerol	AC	1.08 × 10^−4^	78.2 at 0.75 mA cm^−2^	8.8	1110	[33]
chitosan/EMImBF_4_/PEGDA	AC	2.75 × 10^−2^	57.5 at 0.5 Ag^−1^	7.99	250.12	This work

## Data Availability

Not applicable.
